# Patients’ considerations of time toxicity when assessing cancer treatments with marginal benefit

**DOI:** 10.1093/oncolo/oyae187

**Published:** 2024-07-23

**Authors:** Arjun Gupta, Michael D Brundage, Jacqueline Galica, Safiya Karim, Rachel Koven, Terry L Ng, Jennifer O’Donnell, Julia tenHove, Andrew Robinson, Christopher M Booth

**Affiliations:** Division of Hematology, Oncology, and Transplantation, University of Minnesota, Minneapolis, MN, United States; Cancer Care and Epidemiology, Sinclair Cancer Research Institute, Queen’s University, Kingston, ON K7L3N6, Canada; Cancer Care and Epidemiology, Sinclair Cancer Research Institute, Queen’s University, Kingston, ON K7L3N6, Canada; Tom Baker Cancer Centre, Calgary, AB T2N4N2, Canada; Patient Advocate on behalf of Cancer Care and Epidemiology, Sinclair Cancer Research Institute, Queen’s University, Kingston, ON K7L3N6, Canada; Division of Medical Oncology, University of Ottawa, Ottawa ON K1H8L6, Canada; Cancer Care and Epidemiology, Sinclair Cancer Research Institute, Queen’s University, Kingston, ON K7L3N6, Canada; Cancer Care and Epidemiology, Sinclair Cancer Research Institute, Queen’s University, Kingston, ON K7L3N6, Canada; Cancer Centre of Southeastern Ontario, Kingston General Hospital, Kingston, ON K7L2V7, Canada; Cancer Care and Epidemiology, Sinclair Cancer Research Institute, Queen’s University, Kingston, ON K7L3N6, Canada

**Keywords:** time toxicity, time burdens, patient preferences, progression-free survival

## Abstract

**Background:**

Effective techniques for eliciting patients’ preferences regarding their own care, when treatment options offer marginal gains and different risks, is an important clinical need. We sought to evaluate the association between patients’ considerations of the time burdens of care (“time toxicity”) with decisions about hypothetical treatment options.

**Methods:**

We conducted a secondary analysis of a multicenter, mixed-methods study that evaluated patients’ attitudes and preferences toward palliative-intent cancer treatments that delayed imaging progression-free survival (PFS) but did not improve overall survival (OS). We classified participants based on if they spontaneously volunteered one or more consideration of time burdens during qualitative interviews after treatment trade-off exercises. We compared the percentage of participants who opted for treatments with no PFS gain, some PFS gain, or who declined treatment regardless of PFS gain (in the absence of OS benefit). We conducted narrative analysis of themes related to time burdens.

**Results:**

The study cohort included 100 participants with advanced cancer (55% women, 63% age > 60 years, 38% with gastrointestinal cancer, and 80% currently receiving cancer-directed treatment. Forty-six percent (46/100) spontaneously described time burdens as a factor they considered in making treatment decisions. Participants who mentioned time (vs not) had higher thresholds for PFS gains required for choosing additional treatments (*P* value .004). Participants who mentioned time were more likely to decline treatments with no OS benefit irrespective of the magnitude of PFS benefit (65%, vs 31%). On qualitative analysis, we found that time burdens are influenced by several treatment-related factors and have broad-ranging impact, and illustrate how patients’ experiences with time burdens and their preferences regarding time influence their decisions.

**Conclusions:**

Almost half of participating patients spontaneously raised the issue of time burdens of cancer care when making hypothetical treatment decisions. These patients had notable differences in treatment preferences compared to those who did not mention considerations of time. Decision science researchers and clinicians should consider time burdens as an important attribute in research and in clinic.

## Teaser

Effective techniques for eliciting patients’ preferences regarding their own care, when treatment options offer marginal gains, is an important clinical need. This article evaluates the association between patients’ considerations of the time burdens of care (“time toxicity”) with decisions about hypothetical treatment options.

**Implications for Practice**

In treatment trade-off exercises that evaluated patients’ attitudes and preferences toward palliative-intent cancer treatments that delayed imaging progression-free survival (PFS) but did not improve overall survival (OS), participants who spontaneously mentioned considerations of time as a factor in decision-making were more likely to decline treatments with no OS benefit irrespective of the magnitude of PFS benefit (65%, vs 31%). Decision science researchers and clinicians should consider time burdens as an important attribute in research and in clinic.

## Introduction

Patients with advanced cancer and their oncologists face important and sometimes difficult decisions when considering whether to pursue cancer treatments with marginal benefits. Increasingly, especially in the setting of later line systemic treatment for advanced solid cancers, the overall survival benefit of specific cancer treatments can be modest, absent, or unknown (ie, a clinical trial demonstrating an increase in progression-free interval with no report on overall survival).^1,2^ We have previously demonstrated significant heterogeneity in patient preferences regarding accepting a treatment that is known to have progression-free survival (PFS) benefit but no overall survival (OS) benefit.^3^ Thus, for informed shared decision-making, oncologists must elicit patient preferences, communicate data about treatment options, and help patients make decisions that best align with their values.^4^ An overburdened oncology workforce and limited time for patient encounters often constrains how much clinicians can learn about patients’ preferences during clinic encounters.^5^

Strategies that promote shared decision-making, when patients partner with clinicians to integrate their individual values and preferences when making decisions, are needed.^4^ An important first step in developing such strategies is recognizing those factors that may influence patients’ choices. Recently, the time burdens of cancer care for patients and care partners have come into focus, and have been characterized as “time toxicity”.^6-14^ This construct is most relevant in the context of advanced metastatic disease when life expectancy is limited and time becomes most precious.^12,15,16^ Additionally, time toxicity not only represents time taken away from patients and care partners, but is also associated with declines in physical function and worse survival.^17^ We have previously demonstrated that even when a treatment is associated with an absolute OS benefit, the time spent in pursuing, receiving, and recovering from that treatment can be similar in magnitude to the survival time gain.^18^ We have called on oncology clinical trials to report not only survival outcomes, but also where and how patients spend that time, so oncologists can better guide patients regarding treatment choices that align with patients’ goals.^8,19^ In prior and ongoing mixed-methods work with patients and care partners, we have found significant heterogeneity in how individuals perceive time spent in cancer-related activities.^3^ For example, some find extra visits with their clinicians comforting, while others want to avoid even a monthly visit, if possible. Thus, time-related considerations may be an important attribute for patients considering treatments with marginal disease control benefit.

In this study, we conducted a secondary analysis of a recently completed study to determine if an easily evaluable patient expression—the spontaneous and unprompted consideration of the expected time burdens of cancer care—was associated with treatment preferences regarding treatments with marginal benefit. We additionally sought to determine if the mention of time burdens was associated with sociodemographic and clinical characteristics, and to qualitatively examine how patients considered different aspects of time burdens.

## Methods

### Study background, procedures, and primary results

This is a secondary analysis of a multicenter, mixed-methods study that evaluated patients’ attitudes and preferences toward palliative-intent cancer treatments that delayed imaging PFS with no improvement in OS. The study recruited patients with advanced solid cancer who had already received systemic cancer-directed treatment in one of 4 Canadian cancer centers. A total of 100 participants were recruited and met with a research associate, as described in previously.^3^ Participants were asked to consider a hypothetical scenario of a patient with an incurable cancer originating in the abdomen. Treatment trade-off exercises were conducted using pair wise presentations of 2 cancer-directed treatments, one representing standard of care for the hypothetical scenario and the second including additional systemic treatment in 3-weekly cycles. As designed, the strategies had different PFS, treatment-related toxicity, and expected quality-of-life and symptom implications, but overall survival was kept the same (24 months for both strategies). Participants were asked to choose between treatments they would pursue. To identify thresholds that might impact decisions, a sliding bar was used to alter the PFS difference (ie, PFS gains afforded by the additional treatment). To identify how potential OS improvement affect decisions, the sliding bar exercise was repeated with OS gains afforded by additional treatment (keeping previous treatment attributes unchanged). We provide a brief overview of results of the original study here, since the outcome classification in the current study utilizes the classification of participants’ PFS preferences used in the main study. Among the 100 participants, the PFS trade-off exercises^3^ identified 4 groups of participants when assessing treatments with no OS gain: those that prefer treatment with no PFS gain (17%), prefer treatment with some PFS gain (28%), decline treatment regardless of PFS gain, that is, “no threshold” (47%), and unsure of choice (8%). Similarly, the primary OS trade-off exercises identified 4 groups when assessing treatments with OS gain: prefer treatment with 2-month OS gain (42%), prefer treatment with more than 2-month OS gain (42%), decline treatment regardless of OS gain (11%), and unsure (5%).^3^ After each trade-off exercise, the research associate asked participants an open-ended question as to what factors the participant considered, and to share any personal experiences or goals that guided their decision. The research associate did not provide any specific prompts. Members of the research team trained in qualitative methods transcribed interview recordings, and conducted qualitative analyses as described below.

### Exposure, outcome, and covariates

For this secondary analysis, we classified participants into 2 groups based on if they spontaneously volunteered (ie, without specific prompting about time burdens) one or more consideration of time burdens during qualitative interviews (exposure; spontaneously mention time burdens as a consideration or not). The primary outcomes in the current study were the treatment preference with respect to PFS with no OS gain (divided into the same 4 categories as the original study). The secondary outcomes were treatment preferences with respect to OS gain. Covariates included age, sex, education, marital status, employment status, primary cancer site, and type of last systemic therapy.

### Statistical analysis

We used descriptive statistics to provide an overview of sociodemographic and clinical characteristics, and to report the percentage of participants spontaneously considering time toxicity. We tested the association between participants spontaneously considering time toxicity with their categorical PFS and OS treatment preferences using chi-square tests. We further assessed if spontaneous consideration of time toxicity was associated with patient sociodemographic and clinical attributes using chi-square tests. We set statistical significance at *P* < .05.

### Qualitative analysis—identifying the “time toxicity” theme

The original qualitative interviews were exploratory and open-ended without specific prompts—the interviewer did not prompt participants to discuss time. These qualitative findings thus reflect spontaneous reflections from patients about what attributes they consider when making treatment choices, and what matters to them. A team member (J.T.H.) familiar with the original qualitative analysis first reviewed interview transcripts and identified data related to time burdens. Two authors (A.G. and M.D.B.) used reflective thematic analysis, which takes advantage of the researcher’s expertise, experience, and skills, to develop themes from the coded data.^20^ First, they familiarized themselves with the data, separately coded the data, and generated initial themes from the coded and collated data. They then together reviewed, refined, and named themes, which we provide along with exemplar data quotations. In reflexive thematic analysis, researcher subjectivity regarding context is conceptualized as a resource for knowledge production.^21^ In this report, we focus on themes relating to time burdens.

This study was approved by the Research Ethics Board of each cancer center.

## Results

The current analysis included all 100 participants in the primary study: 55% women, 63% >60 years age, 38% with gastrointestinal cancer, and 80% currently receiving cancer-directed treatment. Table 1 lists the sociodemographic and clinical characteristics of the study participants, as well as their classification according to spontaneous mention of time toxicity. Further details regarding participant characteristics are available in the prior publication.^3^ Of the 100 participants, 46 (46%) spontaneously considered time burdens during trade-off exercises. We did not find statistically significant associations between sociodemographic or clinical variables and participants who did and did not spontaneously consider time burdens.

**Table 1.** Sociodemographic and clinical characteristics of participants with advanced cancer making treatment decisions, by whether or not they spontaneously considered time burdens.

Sociodemographic or clinical variable	Participants, number (%)			*P* value
	All (*n* = 100)	Mention time burdens (*n* = 46)	Do not mention time burdens (*n* = 54)	
Age, years				
≤60	37 (37%)	19 (41%)	18 (33%)	.41
>60	63 (63%)	27 (59%)	36 (67%)	
Sex				
Female	55 (55%)	26 (57%)	29 (54%)	.78
Male	45 (45%)	20 (43%)	25 (46%)	
Education				
High school or less	24 (25%)	9 (20%)	15 (29%)	.29
More than high school	72 (96%)	36 (80%)	36 (71%)	
Marital status				
Living with spouse/partner	70 (73%)	32 (71%)	38 (75%)	.71
Single	26 (27%)	13 (29%)	13 (25%)	
Employment				
Employed	13 (14%)	8 (18%)	5 (10%)	.25
Retired or not currently working	83 (86%)	37 (82%)	46 (90%)	
Primary site of cancer				
Gastrointestinal	38 (38%)	16 (35%)	22 (41%)	.82
Breast	17 (17%)	8 (17%)	9 (17%)	
Other	45 (45%)	22 (48%)	23 (42%)	
Most recent systemic therapy				
Cytotoxic chemotherapy	72 (72%)	36 (78%)	36 (67%)	.20
Other	28 (28%)	10 (22%)	18 (33%)	

Four participants—one who mentioned time burdens, and 3 who did not mention time burdens—are missing data on education, marital status, and employment, and are excluded from percentage calculations.

**Figure 1.**
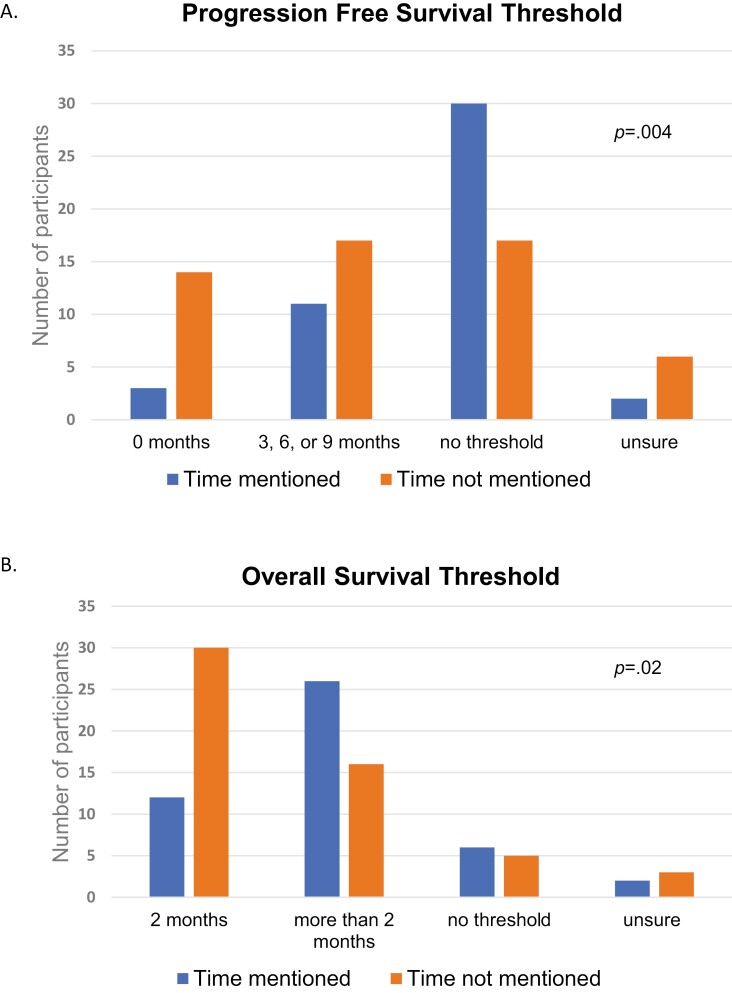
Number of participants (*y*-axis) declaring a preference for additional treatment (threshold) at each treatment benefit level (*x*-axis) by whether or not they spontaneously mentioned time burdens when considering PFS benefits only (no OS benefit, A) and OS benefits (B), respectively.

Figure 1A presents PFS treatment preferences by whether participants mentioned time burdens. Across all participants, those who mentioned time burdens (vs not) had higher PFS thresholds for accepting treatments (*P* = .004). Those who mentioned time burdens were more likely to decline treatments with no OS benefit irrespective of the magnitude of PFS benefit (65%, vs 31%). They were also less likely to opt for treatments with no PFS or OS benefit (7%, vs 26%).

Figure 1B presents OS treatment preferences by whether participants mentioned time burdens. Participants who mentioned time (vs not) had higher OS thresholds for accepting treatments (*P* = .020). For example, participants who mentioned time burdens were less likely to accept treatments with only a 2-month OS benefit (26%, vs 56%).

Reflective thematic analysis of the qualitative data resulted in the identification of 3 main themes, each with constituent categories. First, we found that participants’ perceptions of time burdens were influenced by multiple components. Comments included references to the frequency of visits (“It’s brutal. I mean, every 3 weeks” [participant 079]), the length of visits (“you know that’s a large investment in chair time [p058]), the overall length of treatment (“you are dragging it on for 14 months [p021]), and practical considerations of transport and parking (“time and quality of life is spent trying to find parking” [p011]). Second, the impact of these time burdens goes beyond the lost time. Participants reported a sense of the treatment consuming their lives (“the more you enter into treatment...life becomes cancer and that’s not fun” [p002]), or being tied down (“… you’re tied to the chair… you’re going time after time after time after time.” [p021]). They also noted the impact of treatment time on their overall quality-of-life and psychosocial well-being of themselves and care partners (“a better quality of life is shorter times in treatment” [p014]); “Added days of travel, which is adding more wear and tear to my mental health, and the mental health of everybody around me” [p034]). Third, participants often weighed the investment of treatment-related time against both survival duration (“the amount of investment in time… doesn’t seem like that great of a return.” [p058]) and quality of survival (“I would want to weigh length of time before the cancer comes back against length of treatment and reduction in quality of life.” [p070]). These many perspectives on time toxicity make clear the complexities inherent in providing patients the information they need to meaningfully participate in their treatment decisions. An expanded list of thematic categories and exemplar data is found in Table 2.

**Table 2.** Summary of qualitative themes and subthemes and exemplar quotes regarding time toxicity when considering marginal cancer treatments.

Qualitative theme	Subtheme	Exemplar quote
Time burdens are affected by several factors	Frequency of visits	“Once a month, you know, if it’s going to be really hard on me.” (026) “It’s brutal. I mean, every 3 weeks.” (079)
	Visits can be long	“They have to go through all those additional times where they put the needle in infusions, right?” (033) “Treatment hours are a lot more pervasive.” (008) “Just less time spent at the chemo place.” (038) “You know that’s a large investment in chair time. If that’s a technical term.” (058)
	Overall time on treatment	“ … the length of treatment, 5 months over 14 months… that’s a long time to go through the treatment..” (010) “The length of treatment is really like that’s a year, more than a year. I think I wouldn’t change (to longer treatment). Mostly because it was 6 months instead of 14 months of treatment.” (079) “One (factor) is the length of time, the difference between 4 and 5 months and 13 to 14... It’s a bigger commitment.” (048) “…you are dragging it on for 14 months…” (021)
	Parking and transport	“Time and quality of life is spent trying to find parking.” (011) “ … it’s 36 days of travel, because I have traveled for my cancer treatment, I don’t live directly in the city where a cancer treatment takes place. So that’s 36 lost days.” (034) The lesser (the) amount of treatments, (I have to) travel less days.” (034)
Time burdens have impacts beyond just time	Consuming patients’ lives	“… the more you enter into treatment, the more you are here, the more times you are in a doctor’s appointment or a scanning machine, you know life becomes cancer and that’s not fun.” (002) “Sometimes you just want to scream and say, let me have my life. Give me a few days to do what I want to do.” (048) “You’re spending half your life, half of what life you have, you know, during treatment…” (021)
	Tying patients down	“So on one.. I’m tied up for a whole bunch of months…” (013) “… you’re tied to the chair… you’re going time after time after time after time.” (021) “I would choose the one that would give me more freedom to live my life...” (019)
	Impact on overall quality-of-life	“Just the treatment L lasts longer and going to hospital and all that it’s a very draining experience.” (030) “Treatment L basically has me sitting in a chair every 3 weeks for 14 months … but the quality of life during that time period is significantly reduced.” (058) “A better quality of life is shorter, you know, shorter times (in treatment).” (014) “Feeling like a patient …has a negative impact on quality of life.” (070)
	Psychosocial impact on patients and care partners	“Added days of travel, which is adding more wear and tear to my mental health, and the mental health of everybody around me.” (034) “You know, fewer treatments and you know, less going to the hospital constantly, less feeling like a patient.” (070) “… Psychologically… you’re going time after time after time after time.” (021) “… the continued treatment … a constant reminder of your illness.” (101)
Patients’ experiences with time, preferences regarding time, and expected impact on quantity and quality of time (survival) influence their decisions	Past experiences with time burdens	“I’ve gone through lots of treatments and length of time…” (010).
	Preferences regarding time	“So you might as well be in treatment less time and try to enjoy your life instead of all that extra time and treatment...” (038) “Like I said I am not a fan of hospital.” (030) “I don’t have to go to the hospital all that often, and it makes a big difference, it makes a big difference to me.”(070) “Shorter treatment? Yeah, I would pick M then.” (047)
	Considering impact on how much time (survival) is added	“That’s not a lot of additional months, for an extra looks like quite a few months of treatment.” (028) “It’s a longer treatment schedule, going from over 5 months, versus 15 months and the net gain is only 3 months.” (052) “The amount of investment in time… doesn’t seem like that great of a return.” (058)
	Considering impact of quality of survival time	“So you’ve gained, what 3 months before it returns, but the quality of life, during that time period is significantly reduced.” (058) “I would want to weigh length of time before the cancer comes back against length of treatment and reduction in quality of life.” (070) “So on one I’m tied up for a whole bunch of months, whereas the other is somewhat limited. And the conclusion is going to be the same.” (013)

## Discussion

In this secondary analysis of a multicenter, mixed-methods study that assessed patient preferences regarding treatments for advanced/metastatic cancer that impacted PFS (without OS gains), we found that almost half of participants spontaneously considered the time burdens of the hypothetical cancer care offered when deciding on their treatment preferences. Patients who brought up time burdens were more likely to decline treatments that did not improve OS, regardless of the magnitude of PFS gain. Patients expressed that their considerations of time toxicity were influenced by several treatment-related factors, were guided by their own experiences, and impacted their treatment decisions. By assessing and probing for values and attitudes related to time burdens, clinicians could potentially identify patients who value minimizing treatment (and specifically time) burden. These data also call on clinicians and researchers to specifically consider the time burdens of cancer as an important attribute in decision-making models.

The rate of almost 50% of participants identifying time burdens when considering palliative-intent treatments in the current study is significant. This must be interpreted in the context of the underlying patient population. On one hand, participants were only eligible in the primary study if they had received at least 3 months of systemic cancer-directed treatment for advanced cancer, perhaps allowing patients to experience, and thus be more cognizant of the time burdens of cancer care, versus treatment-naïve patients, as reported by a participant. On the other hand, by requiring participants to have received systemic treatment for at least 3 months, it is plausible we might have selected a population that opted for treatment, potentially excluding patients who declined treatment upfront due to concerns about the time burdens. Thus, the 46% rate may underestimate the frequency with which of this construct is important to patients. Additionally, two-thirds of participants had a gastrointestinal, lung, or brain primary. These patients often experience high symptom burden, treatment complications, and time toxicity (requiring in-person health care contact on one-in-3 or one-in-4 days alive with health care contact),^15^ and thus may be more cognizant of the time burdens of treatment. We did not find that consideration of time burdens varied by patients’ age, education level, or employment status. We similarly did not observe differences by cancer- or treatment-associated features, although our study was not specifically designed to or powered to assess these associations. These results imply that time toxicity is a construct considered by a broad array of patients. The qualitative findings also provide unique insights into patient considerations. Some patients expressed that coming for an appointment once every 3 weeks or every month as “brutal” or “extremely hard”. Patients also described how frequent visits and being tied to a chemotherapy schedule over a length of time can add burdens, interfere with socio-occupational activities, and negatively impact well-being, as represented in a patient quote: “Sometimes you just want to scream and say, let me have my life. Give me a few days to do what I want to do. [p048]” Patients also reported psychosocial distress related to frequent visits for treatment as a constant reminder of being a patient, and burdens care partners.^22^ Interestingly, one patient noted how when accounting for travel time and treatment time, a day with an appointment was a “lost day”. We have highlighted how even purportedly short-clinic appointments can turn into all-day affairs and demonstrated objectively that even simple appointments such as a blood draw can take several hours from patients’ and care partners’ lives.^8^

The primary finding of this study—that patients who declined treatments irrespective of PFS benefit were more likely to voluntarily describe time burdens as a significant variable in their decision-making process—is important for both clinicians and researchers. In clinic, it is imperative for oncology clinicians to quickly and accurately elicit and/or understand patient preferences, especially as more oncology treatments are initially approved on the basis of PFS data.^23,24^ A major barrier to shared decision-making is time pressure faced by oncology clinicians in that typical ambulatory cancer care is plagued by inefficiency and administrative burdens, with little time for sitting down with patients and care partners, to truly have informed shared decision making conversations.^5^ Clinicians can potentially use these findings to elicit patients’ values about time burdens, and assess preferences toward treatments that might only provide PFS benefit. On the research front, decision science, including conjoint analyses techniques such as discrete-choice experiments, can explore the extent to which time burdens contribute to decision making. Several prior discrete-choice experiments assessing patient preferences toward palliative-intent treatments did not consider the time burdens of care, focusing largely on PFS/OS and side effects.^25-28^ Others have considered drug administration attributes (frequency/route/infusion duration, etc.) but have not specifically considered time burdens as a distinct construct.^29,30^ Discrete-choice experiments can also provide data for segmentation techniques such as latent class analysis, which can be used to group participants based on their stated preferences (homogeneous preferences within groups and heterogeneous preferences between groups).^31^ It is also important to note that 17% of patients overall preferred receiving treatments without any PFS or OS benefit—including 7% in patients who mentioned time burdens, and 26% who did not. The 7% rate, which is nonzero, implies that time considerations alone are an insufficient, albeit important, marker of patient preferences. All patients would like to minimize time burdens if given the chance—future work will tease out the importance of this relative to other personal preferences and treatment attributes.

This study has limitations. First, the study cohort included 100 patients from 4 Canadian centers already having opted to receive cancer-directed treatment, and may not be representative across diverse sociodemographic factors and may have excluded patients who declined treatments due to time burdens in the first place. Second, as participants were asked to make treatment choices first, they may have responded to open-ended questions to emphasize and validate their decision made, and may be more likely to describe burdens. Lastly, because this was a secondary analysis, including of qualitative interviews that were exploratory and open-ended, we did not specifically probe patients about specific issues related to time burdens, or how time burdens relate to and interact with other attributes, such as economic, logistic and administrative, care partner, or psychological burdens.^22,32-34^ We are currently conducting focused qualitative analysis that assesses time toxicity more systematically; those data can then be used to inform the most relevant attributes for discrete-choice experiments.

In conclusion, we demonstrate that almost half of participants with advanced cancer spontaneously brought up time burdens associated with a proposed cancer treatment when considering that treatment. Patients who considered time burdens were much more likely to decline a treatment that improved PFS in the absence of OS benefit. These results have the potential to guide clinical practice—oncologists should recognize that patients who bring up time burdens may be less likely to pursue treatments with PFS benefit alone. Decision science researchers should include time burdens and overall time toxicity as a treatment attribute in choice experiments, and explore a group of patients that prioritize minimizing time toxicity.

## Data Availability

The data underlying this article will be shared on reasonable request to the corresponding author.
